# Supplementary material for: Detailed molecular and epigenetic characterization of the pig IPEC-J2 and chicken SL-29 cell lines
Source: iScience. 2023 Feb 20;26(3):106252. doi: 10.1016/j.isci.2023.106252 (PMC10018572; doi:10.1016/j.isci.2023.106252)
Supplement: Data S1. Complete homer output for identified motifs in Pig IPECJ-2, related to Table 2 — Homer motif analysis results for histone modifications H3K4me1, H3K4me3, H3K27ac, and enhancer elements of pig IPECJ2 cell line. P-values >1e-10 are possible false positives. Within each folder (e.g. peak_files_CTCF) are the html files showing the identified motifs when using homer (e.g. homerResults.html). [file mmc2.zip › S5/Pig_IPECJ_2/peak_files_H3K27ac/homerResults/motif2.similar.html]

motif2

## Information for motif2

A
C
T
G
A
G
C
T
C
G
A
T
T
G
C
A
T
A
C
G
A
T
C
G
T
G
A
C
C
T
G
A
  
Reverse Opposite:  

G
A
C
T
A
C
T
G
T
A
G
C
A
T
G
C
A
C
G
T
G
C
T
A
T
C
G
A
T
G
A
C
  

|  |  |
| --- | --- |
| p-value: | 1e-120 |
| log p-value: | -2.768e+02 |
| Information Content per bp: | 1.593 |
| Number of Target Sequences with motif | 26629.0 |
| Percentage of Target Sequences with motif | 50.64% |
| Number of Background Sequences with motif | 23624.2 |
| Percentage of Background Sequences with motif | 45.56% |
| Average Position of motif in Targets | 150.2 +/- 85.0bp |
| Average Position of motif in Background | 150.4 +/- 89.3bp |
| Strand Bias (log2 ratio + to - strand density) | 0.0 |
| Multiplicity (# of sites on avg that occur together) | 1.34 |
| Motif File: | file (matrix) reverse opposite |

### Similar de novo motifs found

|  |  |  |  |  |  |  |  |
| --- | --- | --- | --- | --- | --- | --- | --- |
| Rank | Match Score | Redundant Motif | P-value | log P-value | % of Targets | % of Background | Motif file |
| 1 | 0.665 | A G T C G A C T C G T A T C A G T A C G T A G C G A C T A G C T | 1e-82 | -189.099527 | 42.47% | 38.36% | motif file (matrix) |
| 2 | 0.611 | C A T G T A C G A T G C A C G T C T G A C T A G A T C G A G C T C T G A T G C A | 1e-77 | -177.841534 | 23.95% | 20.59% | motif file (matrix) |
| 3 | 0.650 | G A T C A G C T G C A T T G C A C T A G A T C G T A G C G T A C A G C T A G C T | 1e-62 | -144.757628 | 30.99% | 27.68% | motif file (matrix) |
| 4 | 0.886 | G A C T A T C G A T G C G A T C A G C T T G C A T G C A T G A C C G T A G A T C | 1e-44 | -101.508510 | 12.76% | 10.82% | motif file (matrix) |
| 5 | 0.602 | C G A T G C T A C T G A T C A G G T A C C T G A A G T C G C T A | 1e-41 | -95.463649 | 17.82% | 15.63% | motif file (matrix) |
